# Association of histamine-N-methyl transferase gene polymorphisms with carnosine content in red-brown Korean native chickens

**DOI:** 10.5713/ab.23.0552

**Published:** 2024-04-25

**Authors:** Jean Pierre Munyaneza, Minjun Kim, Eunjin Cho, Aera Jang, Hyo Jun Choo, Jun Heon Lee

**Affiliations:** 1 Division of Animal and Dairy Science, Chungnam National University, Daejeon 34134, Korea; 2 Department of Bio-AI Convergence, Chungnam National University, Daejeon 34134, Korea; 3 Department of Applied Animal Science, College of Animal Life Science, Kangwon National University, Chuncheon 24341, Korea; 4 Poultry Research Institute, National Institute of Animal Science, Pyeongchang 25342, Korea

**Keywords:** Carnosine, *HNMT* gene, Korean native chicken, Meat Flavour, Single-nucleotide Polymorphism (SNP)

## Abstract

**Objective:**

Carnosine and anserine affect the meat flavor. The contents of carnosine and anserine in meat are affected by genetic and environmental factors. This study aimed to discover the single-nucleotide polymorphisms (SNPs) in the histamine-N-methyl transferase (*HNMT*) and histamine-N-methyl transferase-like (*HNMT-like*) genes and to associate them with the content of carnosine and anserine in Korean native chicken-red brown line (KNC-R).

**Methods:**

This study used a total of 384 birds (males, n = 192; females, n = 192) aged 10 weeks old, for genotyping *HNMT* and *HNMT-like* genes. One synonymous SNP (rs29009298C/T) of the *HNMT* gene was genotyped by polymerase chain reaction–restriction fragment length polymorphism (PCR-RFLP) methods whereas four missense SNPs (rs734406537G/A; rs736514667A/G; rs15881680G/A and rs316765035T/C) of the *HNMT* gene, and one missense SNP rs737657949A/C of the *HNMT-like* gene were genotyped by PCR allele competitive extension (PACE) genotyping technology. Two-way analysis of variance of the R program was used to associate *HNMT* genotypes with the contents of carnosine and anserine in KNC-R chickens.

**Results:**

There were significant associations (p<0.05) between the genotypes of the synonymous SNP:rs29009298C/T, missense SNP rs736514667A/G of the *HNMT* gene and the content of carnosine in KNC-Rs. This study also reported the sex effect on the carnosine content, where females had more content of carnosine compared to that of male KNC-R.

**Conclusion:**

Two SNPs (synonymous: rs735769522C/T) and missense: rs736514667A/G) in the *HNMT* gene might be used as genetic markers in the selection and breeding of chickens with better taste and high-flavored meat.

## INTRODUCTION

The selection and breeding of poultry, particularly chickens, with higher growth rates to satisfy the growing demand for poultry meat has resulted in lower meat quality [1]. In contrast, native chickens grow at a slower rate and are known for their superior meat quality [2–4]. After tenderness and juiciness, meat flavor is the most likely trait to be used to describe the quality of the meat during consumption and influences the consumer’s decision to repurchase the meat [5]. Researchers have reported a significant association between flavor precursor content in fresh meat and the final meat flavor [6–13]. These precursors include nucleotides, sugars, amino acids, histidine-containing dipeptides such as carnosine and anserine, organic acids, and fatty acids [9–14] and are responsible for producing the final flavor [11,15]. The red-brown Korean native chicken (KNC-R) is one of the five KNC lines [2,4]. KNC-Rs tend to have a higher body weight and greater content of flavor precursors such as carnosine [2].

The genetic factors (breed or line) and environmental factors such as feed, processing and cooking methods, and storage influence the content of flavor precursors in the meat [10,16, 17]. Previous studies reported candidate genes affecting some of these flavor precursors. For example, *DUSP8* and *IGF2* genes influence the content of nucleotide-related compounds (IMP, inosine, and hypoxanthine) in KNC-Rs [8]. Carnosine is predominantly found in mammals, including beef and pork; by contrast, anserine is abundant in avian species such as poultry [2]. Carnosine is synthesized through the combination of the amino acids β-alanine and L-histidine [18] and is found in large quantities in skeletal muscle, the heart, and the nervous system [19]. Anserine is the methylated product of carnosine [20] and is found in skeletal muscle [21]. Carnosine and anserine are natural antioxidants and have anti-aging and neurotransmitter functions [2]. They also have anti-hypertension properties and contribute to immune boosting and insulin resistance amelioration [22]. Carnosine influences different meat quality traits [23,24]. Additionally, anserine and carnosine confer an umami taste [25].

The flavor of chicken meat is a complex trait and compounds influencing the flavor, including carnosine and anserine, can be controlled genetically [26]; thus, marker-assisted selection to produce highly flavored chicken meat is possible. Moreover, the heritabilities of carnosine and anserine have been reported as moderate for carnosine (0.383) to high (0.531) for anserine in the longissimus muscle of beef [2,23]. The heritabilities of carnosine and anserine were 0.43 and 0.24 in chicken breast meat, respectively [27]. A previous genome-wide association study (GWAS) reported that the genes histamine-N-methyltransferase (*HNMT*) (ENSGALG00000012377) and *HNMT-like* (ENSGALG 00000033461 or LOC771456) likely influence peptide content, including carnosine and anserine contents in KNCs [27]. This is the first work to identify single-nucleotide polymorphisms (SNPs) in *HNMT* and *HNMT-like* genes and investigate their associations with carnosine and anserine contents in chickens. *HNMT* and *HNMT-like* (LOC771456 or carnosine N-methyl transferase 2 [CARNMT2]) have been mapped to chromosome 7 in the chicken genome. *HNMT-like* is thought to be the result of gene duplication, and protein exhibits unique enzymatic activity [28,29]. HNMT and HNMT-like are involved in histidine metabolism [27]. Histidine is bound to alanine to synthesize carnosine in muscle [18,21], and carnosine is then methylated to produce anserine [28,30].

However, polymorphisms in *HNMT* and *HNMT-like* genes are scarce, and their effects on the peptide (carnosine and anserine) contents in chickens are unknown. *HNMT* and *HNMT-like* polymorphisms may affect muscle carnosine and anserine contents, influencing chicken meat flavor. Therefore, our objectives were to identify SNPs in *HNMT* and *HNMT-like* genes and explore possible associations between the identified SNPs and carnosine and anserine contents in KNC-Rs.

## MATERIALS AND METHODS

### Ethical statement

The protocols for animal experiments were approved by the Institution of Animal Care and Use Committee of the National Institute of Animal Science (NIAS; approval number: NIAS 20212219) to ensure the fulfillment of international guidelines for animal welfare.

### Chickens and data collection

The chickens used in this study were kept at the Poultry Research Institute of the NIAS in Pyeongchang, South Korea. Feed and water were provided *ad libitum*. We extracted genomic DNA from 384 birds (males, n = 192; females, n = 192) to identify the SNPs in *HNMT* and *HNMT-like*. All conditions related to the maintenance, management, housing, and feeding of chickens are the same as those stated in our previous work [4]. For the carnosine and anserine analyses, we used the breast meat from 384 KNC-Rs that were slaughtered at 10 weeks of age.

### DNA extraction, primer design, and polymerase chain reaction amplification

Genomic DNA was extracted from 384 KNC-R blood samples using the PrimePrep Genomic DNA Extraction Kit (GenetBio Co., Daejeon, Korea). The primer-BLAST tool of the NCBI database was used to design primer pairs for amplifying the 309-bp fragment of chicken *HNMT* gene (accessed by ENSGALG00000012377 in Ensembl, GRCg6a) containing the synonymous SNP rs29009298C/T. This primer set was synthesized and delivered by Bioneer Corp. (Daejeon, Korea), and are shown in Table 1.

The polymerase chain reaction (PCR) mixture (total volume: 20 μL) comprised 2 μL of genomic DNA (25 ng/μL), 1 μL of forward and reverse primer each, 10 μL of HS Prime Taq Premix (2×) (GenetBio Co., Korea), and 6 μL of triple-distilled water (3DW). PCR amplification was carried out using a T100 Thermal Cycler (Bio-Rad Laboratories, Inc., Hercules, CA, USA) with the following steps: initial denaturation at 95°C for 3 min, followed by 35 cycles of denaturation at 95°C for 30 s, annealing at 63°C for 45 s, extension at 72°C for 60 s, and a final extension step at 72°C for 10 min. The PCR products were separated by electrophoresis in 2% agarose gel stained with ethidium bromide at 120 V for 30 min; the fragments were visualized under an ultraviolet (UV) transilluminator (ATTO Corporation, Tokyo, Japan).

### Next-generation sequencing data analysis

Next-generation sequencing (NGS) data for the KNCs were obtained from the National Agricultural Biotechnology Information Center (Jeonju, Korea) to detect and confirm the SNPs in the *HNMT* and *HNMT-like* genes of the KNC-Rs. Four missense and four synonymous variants in *HNMT* were identified. Additionally, one missense and three synonymous variants were discovered in *HNMT-like*. Here, we focused on one synonymous variant (rs29009298C/T) and all four missense variants (rs734406537G/A, rs736514667A/G, rs15881680G/A, and rs316765035T/C) of *HNMT*. For *HNMT-like*, one missense variant (rs737657949A/C) was used for genotyping.

### Genotyping of *HNMT* and *HNMT-like*

We used the polymerase chain reaction–restriction fragment length polymorphism (PCR-RFLP) genotyping method for the synonymous variant in *HNMT* (rs29009298C/T). PCR allele competitive extension (PACE) genotyping was used to determine the differences in the genetic makeup of missense variants in *HNMT* and *HNMT-like*. For genotyping of the *HNMT* (SNP: rs29009298C/T), we used a total volume of 20 μL composed of 15 μL of PCR product, 0.4 μL of restriction enzyme (*HpyCH4IV*), 2 μL of 10× CutSmart Buffer, and 2.6 μL of 3DW. The mixture was incubated at 37°C for 6 h. The *HpyCH4IV* restriction enzyme was selected using NEBcutter2 software (https://nc2.neb.com/NEBcutter2/).

The RFLP fragments of *HNMT* (rs29009298C/T) digested with the *HpyCH4IV* restriction enzyme were separated using 3% agarose gel electrophoresis stained with ethidium bromide at 90 V for 45 min and visualized using a UV transilluminator (ATTO Corporation, Tokyo, Japan). The PACE assay mix and PACE master mix were produced by 3CR Bioscience (Harlow, UK) to genotype the missense variants in *HNMT* and *HNMT-like* genes (Table 1). PACE genotyping was performed in a 96-well plate. Each well contained a mixture of 10 μL, composed of 1 μL of genomic DNA (5 ng/μL) or 1 μL of 3DW in the case of the negative control, 5 μL of master mix, 0.25 μL of assay mix, and 3.75 μL of 3DW. The reaction was run using the CFX Connect Real-time PCR Detection System (Bio-Rad Laboratories, Inc., USA).

### Analysis of carnosine and anserine contents in KNC-Rs

The carnosine and anserine contents in the breast meat of KNC-Rs were analyzed using nuclear magnetic resonance following a previously described method [31]. The carnosine and anserine contents are expressed as milligrams per 100 grams of breast meat (mg/100 g of breast meat).

### Statistical analyses

After genotyping of *HNMT* in KNC-Rs, the genotype and allele frequencies were calculated according to Nei and Kumar [32], in which the chi-square (χ^2^) test for Hardy–Weinberg equilibrium (HWE) was estimated using the formula proposed in Hartl and Clark [33]. To examine the effects of genotype and sex on carnosine and anserine contents in KNC-Rs, a two-way analysis of variance was conducted using R software ver. 4.2.1 software [34].

The mathematical model is represented by:


$${\text{Y}}_{\text{i},\text{j}}=\mu +{\text{G}}_{\text{i}}+{\text{S}}_{\text{j}}+{ɛ}_{\text{i},\text{j}}$$

where ***Y****_ij_* represents carnosine or anserine content, *μ* is the population mean, *G_i_* represents genotype effects, *S_j_* represents the effects of sex, and *ɛ_i,j_* is the residual error. Significance tests between the mean value of each genotype and the contents of carnosine or anserine were carried out using Tukey’s test (p<0.05).

## RESULTS

### Next-generation sequencing-based identification of single-nucleotide polymorphisms

Analysis of the NGS data of KNC-Rs revealed four missense (rs734406537G/A, rs736514667A/G, rs15881680G/A, and rs316765035T/C) and four synonymous (rs317627831A/G, rs312743068T/C, rs29009298C/T, and rs735769522C/T) variants in *HNMT*, as well as one missense (rs737657949A/C) and three synonymous (rs317607580C/T, rs316762204A/G, and rs316857074A/G) variants in *HNMT-like*.

### Genotyping of *HNMT* and *HNMT-like* single-nucleotide polymorphisms

**Figure 1:**
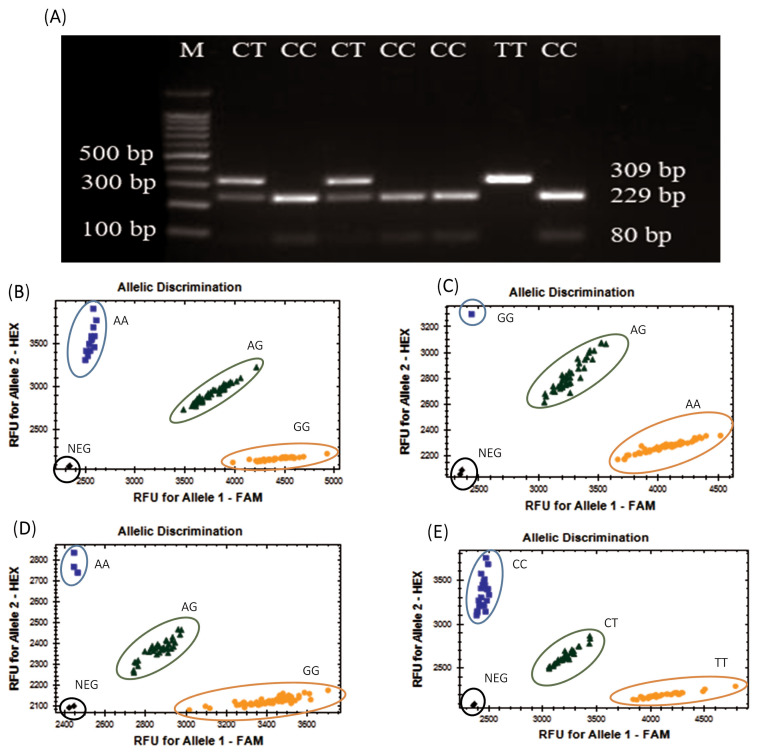
RFLP genotyping results of *HNMT* (SNP rs29009298C/T, synonymous) visualized using 3% agarose gel electrophoresis from samples 1–7 (A); PACE genotyping results for missense variants of *HNMT*: rs734406537G/A (B); rs736514667A/G (C); rs15881680G/A (D); and rs316765035T/C (E). NEG, negative control; RFLP, restriction fragment length polymorphism; HNMT, Histamine-N-methyltransferase; SNP, single-nucleotide polymorphism; PACE, PCR allele competitive extension. DNA marker with 100 bp.

A synonymous SNP (rs29009298C/T) of *HNMT* and missense SNPs in *HNMT* and *HNMT-like* genes were genotyped using PCR-RFLP and PACE genotyping technology, respectively. The 309-bp fragment containing the synonymous SNP in *HNMT* (rs29009298C/T) was successfully amplified and then digested by *HpyCH4IV*, yielding three genotypes: CC, CT, and TT (Figure 1). PACE genotyping of the four *HNMT* missense SNPs (rs734406537G/A, rs736514667A/G, rs15881680G/A, and rs316765035T/C) yielded three genotypes (Figure 1). PACE genotyping of the *HNMT-like* missense SNP rs737657949A/C yielded one genotype, AA.

### Genotype frequencies, allele frequencies, and Hardy–Weinberg equilibrium

For the synonymous SNP: rs29009298C/T of *HNMT* gene, the most frequent genotype was CC, representing 64% of the total genotyped chickens, followed by the heterozygous genotype CT (34%) and TT (2%), and the C allele was the predominant allele (80%) over the allele T (20%). For the missense variant rs734406537G/A, the heterozygous genotype (AG) was the predominant genotype (50%), followed by GG (40%) and AA (10%). For the missense SNP rs73651 4667A/G, the predominant genotype was AA (61%), followed by AG (36%) and GG (3%). For the missense SNP rs15881680G/A, the dominant genotype was GG (59%), followed by AG (36%) and AA (5%). For the missense SNP rs316765035T/C, the dominant genotype was CT (50%), followed by TT (32%) and AA (18%) (Table 2). Finally, the missense SNP rs737657949A/C of the *HNMT-like* gene was monomorphic, with one allele (A) and one genotype (AA) (Table 2).

### Association of *HNMT* genotypes and sex with carnosine and anserine contents in KNC-R

We found significant influences (p<0.05) of the *HNMT* genotypes with the synonymous SNP rs29009298C/T and the missense SNP rs736514667A/G on carnosine content in KNC-Rs (Table 3). Furthermore, we used the PolyPhen-2 v2.2.3r406 [35] biotechnology tool to predict the functional consequence for rs736514667A/G variant which changes the encoded amino acid residue 30 of HNMT gene, replacing glutamine (Gln) by arginine (Arg). The polyphen-2 result reports using 2 different models (HumDiv and HumVar) predicted that the rs736514667A/G variant is probably damaging with a score of 0.991 (sensitivity: 0.71; specificity: 0.97) (HumDiv) and the same variant is possibly damaging with a score of 0.873 (sensitivity: 0.71; specificity: 0.89) (HumVar). These predictions of the functional consequence for rs73651 4667A/G variant confirms that rs736514667A/G might be a reasonable genetic marker for carnosine content in KNC-Rs. Additionally, we observed a significant association (p<0.05) between sex and carnosine content in KNC-Rs; females had higher breast-meat carnosine contents than males (Table 4). Because the genotyping of the *HNMT-like* missense SNP rs737657949A/C yielded a single genotype, AA, it could not be used for association studies.

## DISCUSSION

*HNMT* and *HNMT-like* genes, influence the content of carnosine and anserine, respectively [27]. The present study validated the effects of these candidate genes on the carnosine and anserine contents in KNC-Rs. *HNMT* and *HNMT-like* are involved in histidine metabolism [27]. The content of flavor precursors in raw meat, including carnosine and anserine, influences the flavor of the cooked meat [9–13]. KNC-Rs have higher contents of flavor precursors such as carnosine compared to broilers or other KNC lines [2].

Carnosine is synthesized in muscles from L-histidine and β-alanine via carnosine synthase [20]; it can also be metabolized by carnosinase back into alanine and histidine [18]. HNMT-like catalyzes carnosine methylation to produce anserine [28,30]. Histidine and histamine are two metabolites of carnosine [36]. Histamine is synthesized from histidine via histidine decarboxylase [37] and is then degraded into N-methyl histamine by the enzyme HNMT [37]. Histamine acts as a neurotransmitter [38] and has effects on the immune system [37]. Moreover, histamine is an indicator of meat quality, freshness, and safety [39]. However, high amounts of histamine may be toxic, inducing allergy symptoms such as swelling, rash, hives, diarrhea, and headache [37,39,40]. Excess histamine is metabolized by either HNMT into N-methylhistamine or diamine oxidase into imidazole acetaldehyde [40,41]. Therefore, HNMT has a key role in histamine homeostasis to prevent the consequences of accumulated histamine in the body.

Genotyping of the *HNMT* gene for all five SNPs (one synonymous: rs29009298C/T; four missense: rs734406537G/A, rs736514667A/G, rs15881680G/A, and rs316765035T/C) yielded three genotypes (Table 2). Based on the χ^2^ results, all SNPs of *HNMT* were in HWE. This may be explained by the absence of evolutionary drivers in the sampled population [42]. Additionally, selection of KNC-Rs may have led to the fixation of one allele (A) for the missense SNP rs737657949A/C of *HNMT-like*. KNC-Rs with the homozygous genotype CC of the synonymous SNP rs29009298C/T had higher carnosine contents compared to those with the genotypes CT and TT. Moreover, KNC-Rs with the homozygous genotype AA had higher carnosine contents compared to those with the genotypes AG and GG for the *HNMT* missense SNP rs736514 667A/G. We also found a significant effect of sex on carnosine content in KNC-Rs; female chickens had higher carnosine contents in breast meat compared to males (Table 4). These results agreed with those of previous researchers who reported higher carnosine contents in female chickens compared with male chickens [2,43].

Carnosine influences meat quality traits such as redness and drip loss [24]. Moreover, carnosine and anserine are reportedly associated with the umami taste [25]. Thus, identifying SNPs and genotypes associated with higher carnosine and anserine contents is of major importance in the selection and breeding of chickens with desirable flavor qualities, which are typically related to healthier, stronger flavored meat. The limitation of our study was small sample size. Thus, the results (identification of the SNPs in *HNMT* gene and their association with the content of carnosine in KNC-Rs) of the current study must be validated in larger sample sizes of different populations, lines and chicken breeds.

## CONCLUSION

Carnosine and anserine are one of the flavor precursors which affect the taste and flavor of the meat. Carnosine and anserine confer various health benefits to meat consumers. Our results confirmed the genetic effects of carnosine and identified SNPs in the *HNMT* gene of KNC-Rs that affect breast meat carnosine content. The homozygous genotype CC of the synonymous SNP rs29009298C/T was associated with a higher carnosine content compared to the genotypes CT and TTwhile genotype AA has a higher carnosine content compared to the genotypes AG and GG for the *HNMT* missense SNP rs736514667A/G. Moreover, the meat of female KNC-Rs contained higher carnosine contents compared to male chickens. The synonymous SNP rs29009298C/T and the missense SNP rs736514667A/G of the *HNMT* gene might be considered as potential genetic markers for the breeding of chickens with stronger flavored meat.

## Figures and Tables

**Table 1:** Primers, genes, SNPs, PCR product (bp), annealing temperature, and genotyping method

Gene	SNP/location	Primer F/R	Amplicon (bp)	Annealing temperature (°C)	Genotyping method
*HNMT*	rs29009298C/T (synonymous)	TATCCCTCAGAAACCAGTGG GTTCTCACTGCATCCAGGCT	309	63	RFLP
	rs734406537G/A (missense)	Forward primer X, Y (5’-3’) GAAGGTGACCAAGTTCATGCTGCTGACAGACCTCAGCCG/ GAAGGTCGGAGTCAACGGATTCCTGCTGACAGACCTCAGCCA Common primer AGACGTGGAAGCAGCGGACGTA G/A (FAM/HEX)	NA	55	PACE
	rs736514667A/G (missense)	Forward primer X, Y (5’-3’) GAAGGTGACCAAGTTCATGCTGGCCAAGTCTACTGAGCACCA/ GAAGGTCGGAGTCAACGGATTGGCCAAGTCTACTGAGCACCG Common primer CTGCTGCTCCACAAACTGCTTCAT A/G (FAM/HEX)	NA	55	PACE
	rs15881680G/A (missense)	Forward primer X, Y (5’-3’) GAAGGTGACCAAGTTCATGCTAGGAAAGGAAGACCCATACTTCC/ GAAGGTCGGAGTCAACGGATTGTAGGAAAGGAAGACCCATACTTCT Common primer CCTCAGAAACCAGTGGCTGGGAA G/A (FAM/HEX)	NA	55	PACE
	rs316765035T/C (missense)	Forward primer X, Y (5’-3’) GAAGGTGACCAAGTTCATGCTACAGATTCCTGCATTAGGAGACCTT/ GAAGGTCGGAGTCAACGGATTAGATTCCTGCATTAGGAGACCTC Common primer CACATCACTGTAGCAGCTTATAGATGTA T/C (FAM/HEX)	NA	55	PACE
*HNMT-like*	rs737657949A/C (missense)	Forward primer X, Y (5’-3’) GAAGGTGACCAAGTTCATGCTAGATGCTGTACCGTGTGGAAGATA/ GAAGGTCGGAGTCAACGGATTGATGCTGTACCGTGTGGAAGATC Common primer CTGTGGAAAAACTTGATGGTGTTAGGAA A/C (FAM/HEX)	NA	55	PACE

SNPs, single-nucleotide polymorphisms; PCR, polymerase chain reaction; RFLP, restriction fragment length polymorphism; NA, not applicable; PACE, polymer chain reaction allele competitive extension.

**Table 2:** Genotype and allele frequencies, and HWE for *HNMT* and *HNMT-like* genes in KNC-Rs

Gene	SNP	Sample size	Genotype frequency			Allele frequency		χ^2^ calc.^1)^
*HNMT*	rs29009298C/T (synonymous)	384	CC (244)	CT (131)	TT (9)	C	T	3.2
			0.64	0.34	0.02	0.80	0.20	
	rs734406537G/A (missense)	384	GG (155)	AG (194)	AA (35)	G	A	1.2
			0.40	0.50	0.10	0.66	0.34	
	rs736514667A/G (missense)	384	AA (234)	AG (138)	GG (12)	A	G	2.45
			0.61	0.36	0.03	0.79	0.21	
	rs15881680G/A (missense)	384	GG (225)	AG (139)	AA (20)	G	A	0.1
			0.59	0.36	0.05	0.77	0.23	
	rs316765035T/C (missense)	384	TT (122)	CT (192)	CC (70)	T	C	0.15
			0.32	0.50	018	0.57	0.43	
*HNMT-like* (LOC771456)	rs737657949A/C (missense)	384	AA	AC	CC	A	C	NA
			100	0	0	100	0	

Individuals with specific genotypes are shown in parentheses.

HWE, Hardy–Weinberg equilibrium; HNMT, histamine-N-methyltransferase; KNC-R, red-brown Korean native chicken; NA, not applicable.

1) χ^2^ calc., chi-square calculated; χ^2^ table (p<0.05) = 3.84.

**Table 3:** Association of *HNMT* genotypes with carnosine and anserine contents in KNC-Rs

Gene	SNP	Trait (mg/100 g)	Genotypes		
			CC (244)	CT (131)	TT (9)
			
*HNMT*	rs29009298C/T (synonymous)	Carnosine	283.44 ±56.3^a^	270.17±56.4^b^	238.28±34.78^b^
		Anserine	721.60±74.9	739.80±69.02	741.27±62.77
			
			GG (155)	AG (195)	AA (35)
			
	rs734406537G/A (missense)	Carnosine	279.34±60.91	277.60±57.32	262.37±51.86
		Anserine	723.27±84.50	736.87±71.55	752.97±75.32
			
			AA (234)	AG (138)	GG (12)
			
	rs736514667A/G (missense)	Carnosine	282.53±56.54^a^	267.04±56.65^b^	234.26±46.22^b^
		Anserine	728.24±81.62	735.25±67.88	769.18±64.55
			
			GG (225)	AG (139)	AA (20)
			
	rs15881680G/A (missense)	Carnosine	274.90±55.57	276.34±55.02	272.22±76.99
		Anserine	726.62±78.57	733.86±81.02	738.22±64.65
			
			TT (122)	CT (192)	CC (70)
			
	rs316765035T/C (missense)	Carnosine	277.81±57.22	278.28±59.40	267.79±50.76
		Anserine	727.99±83.74	732.87±73.33	738.26±67.77

Individuals with specific genotypes are shown in parentheses.

The contents of carnosine and anserine are expressed as mg/100 g breast meat.

*HNMT*, histamine-N-methyltransferase; KNC-R, red-brown Korean native chicken; SNPs, single-nucleotide polymorphisms.

a,b Means within a row with different superscripts differ significantly at p<0.05.

**Table 4:** Effects of sex on carnosine and anserine contents in KNC-Rs

Sample size	Trait	Sex	

		Male (n = 192)	Female (n = 192)
384	Carnosine	269.41±57.60^b^	**286.11±54.42** ^a^
	Anserine	721.16±65.50	735.67±79.43

Individuals with specific genotypes are shown in parentheses.

The contents of carnosine and anserine are expressed as mg/100 g breast meat.

KNC-R, red-brown Korean native chicken.

a,b Means within a row with different superscripts differ significantly at p<0.05.

## References

[1] Petracci M, Cavani C (2012). Muscle growth and poultry meat quality issues. Nutrients.

[2] Jung S, Bae YS, Kim HJ (2013). Carnosine, anserine, creatine, and inosine-5′-monophosphate contents in breast and thigh meats from 5 lines of Korean native chicken. Poult Sci.

[3] Cahyadi M, Park HB, Seo DW (2020). Association of the thyroid hormone responsive spot 14 alpha gene with growth-related traits in Korean native chicken. Asian-Australas J Anim Sci.

[4] Kim M, Munyaneza JP, Cho E (2023). Genome-wide association study on the content of nucleotide-related compounds in Korean native chicken breast meat. Animals.

[5] Maltin C, Balcerzak D, Tilley R, Delday M (2003). Determinants of meat quality: tenderness. Proc Nutr Soc.

[6] Khan MI, Jo C, Tariq MR (2015). Meat flavor precursors and factors influencing flavor precursors-a systematic review. Meat Sci.

[7] Ismail I, Joo ST (2017). Poultry meat quality in relation to muscle growth and muscle fiber characteristics. Korean J Food Sci Anim Resour.

[8] Munyaneza JP, Kim M, Cho E, Jang A, Choo HJ, Lee JH (2023). Association of single-nucleotide polymorphisms in dual specificity phosphatase 8 and insulin-like growth factor 2 genes with inosine-5′-monophosphate, inosine, and hypoxanthine contents in chickens. Anim Biosci.

[9] Sasaki K, Motoyama M, Mitsumoto M (2007). Changes in the amounts of water-soluble umami-related substances in porcine longissimus and biceps femoris muscles during moist heat cooking. Meat Sci.

[10] Jayasena DD, Ahn DU, Nam KC, Jo C (2013). Factors affecting cooked chicken meat flavor: a review. Worlds Poult Sci J.

[11] Jayasena DD, Kim SH, Lee HJ (2014). Comparison of the amounts of taste-related compounds in raw and cooked meats from broilers and Korean native chickens. Poult Sci.

[12] Hu J, Yu P, Ding X, Xu M, Guo B, Xu Y (2015). Genetic polymorphisms of the AMPD1 gene and their correlations with IMP contents in Fast Partridge and Lingshan chickens. Gene.

[13] Uemoto Y, Ohtake T, Sasago N (2017). Effect of two non-synonymous ecto-5′-nucleotidase variants on the genetic architecture of inosine 5′-monophosphate (IMP) and its degradation products in Japanese Black beef. BMC Genomics.

[14] Jin S, Park HB, Seo D (2018). Identification of quantitative trait loci for the fatty acid composition in Korean native chicken. Asian-Australas J Anim Sci.

[15] Shahidi F, Hossain A (2022). Role of lipids in food flavor generation. Molecules.

[16] Chumngoen W, Tan FJ (2015). Relationships between descriptive sensory attributes and physicochemical analysis of broiler and Taiwan native chicken breast meat. Asian-Australas J Anim Sci.

[17] Ma T, Xu L, Wang H (2015). Mining the key regulatory genes of chicken inosine 5′-monophosphate metabolism based on time series microarray data. J Anim Sci Biotechnol.

[18] Boldyrev AA, Aldini G, Derave W (2013). Physiology and pathophysiology of carnosine. Physiol Rev.

[19] Forsberg EA, Botusan IR, Wang J (2015). Carnosine decreases IGFBP1 production in db/db mice through suppression of HIF-1. J Endocrinol.

[20] Blancquaert L, Baba SP, Kwiatkowski S (2016). Carnosine and anserine homeostasis in skeletal muscle and heart is controlled by β-alanine transamination. J Physiol.

[21] Wu G (2020). Important roles of dietary taurine, creatine, carnosine, anserine and 4-hydroxyproline in human nutrition and health. Amino Acids.

[22] Teeravirote K, Sutthanut K, Thonsri U (2022). Anserine/Carnosine-rich extract from Thai native chicken suppresses melanogenesis via activation of ERK signaling pathway. Molecules.

[23] D'Astous-Pagé J, Gariépy C, Blouin R (2017). Identification of single nucleotide polymorphisms in carnosine-related genes and effects of genotypes on pork meat quality attributes. Meat Sci.

[24] Ma XY, Jiang ZY, Lin YC, Zheng CT, Zhou GL (2010). Dietary supplementation with carnosine improves antioxidant capacity and meat quality of finishing pigs. J Anim Physiol Anim Nutr (Berl).

[25] Dashdorj D, Amna T, Hwang I (2015). Influence of specific taste active components on meat flavor as affected by intrinsic and extrinsic factors: an overview. Eur Food Res Technol.

[26] Zhang L, Hao Z, Zhao C (2021). Taste compounds, affecting factors, and methods used to evaluate chicken soup: a review. Food Sci Nutr.

[27] Kim M, Munyaneza JP, Cho E (2024). Genome-wide association studies of anserine and carnosine contents in the breast meat of Korean native chickens. Poult Sci.

[28] Drozak J, Chrobok L, Poleszak O, Jagielski AK, Derlacz R (2013). Molecular identification of carnosine N-methyltransferase as chicken histamine N-methyltransferase-like protein (hnmt-like). PLoS One.

[29] Kwiatkowski S, Kiersztan A, Drozak J (2018). Biosynthesis of carnosine and related dipeptides in vertebrates. Curr Protein Pept Sci.

[30] Andersen SM, Waagbø R, Espe M (2016). Functional amino acids in fish health and welfare. Front Biosci (Elite Ed).

[31] Kim HC, Ko YJ, Jo C (2021). Potential of 2D qNMR spectroscopy for distinguishing chicken breeds based on the metabolic differences. Food Chem.

[32] Nei M, Kumar S (2000). Molecular evolution and phylogenetics.

[33] Hartl DL, Clark AG (1997). Principle of population genetics.

[34] R Core Team (2022). R: a language and environment for statistical computing [Internet].

[35] Adzhubei IA, Schmidt S, Peshkin L (2010). A method and server for predicting damaging missense mutations. Nat Methods.

[36] Dai YJ, Wu DC, Feng B (2015). Protective effect of carnosine on febrile seizures in immature mice. Neurosci Lett.

[37] Huang H, Li Y, Liang J, Finkelman FD (2018). Molecular regulation of histamine synthesis. Front Immunol.

[38] Wójcik W, Łukasiewicz-Mierzejewska M, Damaziak K, Bień D (2022). Biogenic amines in poultry meat and poultry products: formation, appearance, and methods of reduction. Animals.

[39] Michalski M, Pawul-Gruba M, Madejska A (2021). Histamine contents in raw long-ripening meat products commercially available in Poland. J Vet Res.

[40] Maintz L, Novak N (2007). Histamine and histamine intolerance. Am J Clin Nutr.

[41] Neumann J, Grobe JM, Weisgut J (2021). Histamine can be formed and degraded in the human and mouse heart. Front Pharmacol.

[42] Allendorf FW, Luikart G, Aitken SN (2013). Conservation and genetics of populations.

[43] Suwanvichanee C, Sinpru P, Promkhun K (2022). Effects of b-alanine and L-histidine supplementation on carnosine contents in and quality and secondary structure of proteins in slow-growing Korat chicken meat. Poult Sci.

